# National Cross-Sectional Study Assessing the Positivity Rate and Clinical Manifestations of Human Bocavirus Respiratory Infections Among Hospitalized Children Under 5 Years of Age in Jordan

**DOI:** 10.3390/pathogens15050515

**Published:** 2026-05-12

**Authors:** Mohammad Abu Lubad, Munir Abu-Helalah, Mohammad Al-Hanaktah, Maisalreem Alhousani, Jana Khatatbeh, Ahmad Al Tibi, Amin Aqel, Simon B. Drysdale

**Affiliations:** 1 Department of Microbiology and Pathology, Faculty of Medicine, Mutah University, Karak 61710, Jordan; abu_lubbad@mutah.edu.jo (M.A.L.);; 2 Faculty of Medicine, Aqaba Medical Sciences University, Aqaba 77110, Jordan; 3 Department of Family and Community Medicine, Faculty of Medicine, The University of Jordan, Amman 11942, Jordan; 4 Public Health Institute, The University of Jordan, Amman 11942, Jordan; 5 Faculty of Medicine, The University of Jordan, Amman 11942, Jordan; malhanaktah49@gmail.com (M.A.-H.); khtatbehjana3@gmail.com (J.K.); 6 Faculty of Medicine, Mutah University, Karak 61710, Jordan; maishousani@gmail.com; 7 Molecular Genetics Department, Biolab Diagnostic Laboratories, P.O. Box 5153, Amman 11183, Jordan; a.tibi@biolab.jo; 8 Oxford Vaccine Group, Department of Pediatrics, University of Oxford, Oxford OX1 2JD, UK; simon.drysdale@paediatrics.ox.ac.uk; 9 The NIHR Oxford Biomedical Research Centre, Oxford OX3 7JX, UK

**Keywords:** human bocavirus, clinical, epidemiological, children, below age of five, Jordan

## Abstract

Human bocavirus (HBoV) is an important respiratory pathogen in young children, but recent data from Jordan are limited. This study assessed the positivity rate, epidemiological profile, clinical manifestations, and predictors of HBoV infection among hospitalized children aged <5 years in Jordan. In this national multicenter cross-sectional study, 1000 children aged <5 years admitted with acute respiratory infection to four hospitals in Jordan between November 2022 and March 2023 were included. Nasopharyngeal specimens were tested for HBoV, and demographic, clinical, and hospitalization data were analyzed using descriptive statistics and logistic regression. HBoV was detected in 48/1000 children (4.8%), with the highest positivity in January 2023 (15.5%). Fever (100%), cough (100%), rhinorrhea (56.3%), respiratory crackles (58.3%), and breathlessness (47.9%) were the most frequent manifestations. Pneumothorax/atelectasis was more frequent in HBoV-positive than HBoV-negative children (2.1% vs. 0.2%; *p* = 0.021). Household smoking, residence outside Amman, and longer hospital stay were independent predictors of HBoV positivity. HBoV infection was not independently associated with complications. HBoV accounted for a measurable proportion of pediatric respiratory hospitalizations in Jordan and remains a relevant contributor to respiratory morbidity in children under 5 years.

## 1. Introduction

Human bocavirus (HBoV) is a DNA virus that belongs to the *Parvoviridae* family. It was documented for the first time in 2005 in children with respiratory tract infection [1]. According to Manning et al., the majority of bocavirus cases were children (6–24 months old), with peak incidence in midwinter months [2]. HBoV is found to be associated with respiratory and gastrointestinal diseases. It can be detected as a single infection or in combination with other pathogens, with 80% of hospitalized children showing co-infections with other viruses, most commonly with rhinovirus, which was reported in up to 45% of cases [3]. In addition to viral pathogens, bacterial organisms were identified in association with HBoV, most frequently with *Streptococcus pneumoniae* [3]. Children with co-infections often experience more severe illnesses that may require intensive care. However, bocavirus infection alone can be potentially fatal, particularly in lower respiratory tract infections, with pneumonia as the most common manifestation [4].

Studies have reported a varying prevalence depending on region and population. A survey conducted in the United States found that human bocavirus was detected in 5.2% of children, with most cases occurring in those under 2 years of age. Specimens in this study were obtained from children seen in emergency departments, inpatient wards, intensive care units, and hospital-affiliated outpatient urgent care clinics. Furthermore, more than 50% of the infected children presented with respiratory symptoms [5]. The PERCH multi-country case-control study included nine sites in seven countries: Bangladesh, The Gambia, Kenya, Mali, South Africa, Thailand, and Zambia. This study showed that human bocavirus was positive among 13.1% of children aged 1–59 months admitted to the hospital with severe pneumonia [6]. Regionally, a systematic review of the Middle East and North Africa (MENA) region showed a wide variation in prevalence among children, ranging from 0% in Iran to 56.8% in Egypt [7].

In Jordan, HBoV was detected in 57 of 312 hospitalized children under the age of 5 years with acute respiratory infection between December 2003 and May 2004. Detection rates were higher in severe cases compared with mild or moderate disease [8]. Another study found that more than half of those affected were infants younger than 6 months, and the main symptoms were cough, runny nose, and fever. Co-infections were associated with more severe disease, including more respiratory distress and diarrhea [9].

Although HBoV has been detected in various countries, data from Jordan remain limited, and most prior studies have focused on clinical surveillance rather than on epidemiology, seasonality, and clinical predictors in the post-COVID-19 era. The present study is part of a larger national multi-center surveillance program of respiratory pathogens in hospitalized Jordanian children under five years of age, originally established to evaluate the burden of respiratory syncytial virus (RSV), influenza, and SARS-CoV-2 [10]. Using the same prospective cohort, the same nasopharyngeal specimens, and the same case report form, our group has subsequently conducted pre-planned virus-specific secondary analyses for human adenovirus (HAdV) [11] and human parainfluenza viruses (HPIV) [12]. Each report addresses a distinct respiratory pathogen with its own epidemiological, clinical, and public health considerations, and together they form a coordinated, transparent series of analyses derived from the same surveillance dataset. The current report is the next pathogen-specific output of this program and examines the positivity rate, epidemiological profile, clinical manifestations, complications, and predictors of HBoV infection in the same cohort [10,11,12,13].

## 2. Materials and Methods

### 2.1. Study Design and Related Work

The present manuscript is a pre-planned, virus-specific secondary analysis of specimens prospectively collected as part of a larger national multi-center surveillance program on respiratory viral infections in Jordanian children under five years of age. The parent study, including its sampling frame, eligibility criteria, specimen handling, and case report form, has been described in detail previously [10]. Virus-specific analyses of the same cohort have been published for respiratory syncytial virus [10], human adenovirus [11], influenza virus [13], and human parainfluenza viruses [12]. The current report addresses human bocavirus (HBoV). The cohort, the nasopharyngeal specimens, the laboratory workflow, and the demographic and clinical case-report-form data are shared across these reports, but no participant-level primary outcome is duplicated. This disclosure is provided to ensure full transparency for readers and to allow appropriate handling of cohort overlap in any future systematic reviews or meta-analyses that include data from this surveillance program.

### 2.2. Study Population

Study participants included children under five years of age admitted to the designated clinical sites who met the following eligibility criteria: (1) clinical evidence of acute infection, indicated by a core temperature of ≥38 °C or <35.5 °C, an abnormal white blood cell (WBC) count, or an abnormal differential; and (2) a diagnosis of acute respiratory infection as previously defined [1]. Conversely, children who were not permanent residents of Jordan were excluded from the study.

### 2.3. Microbiology

This study involved the secondary analysis of previously collected specimens, which were stored for 18 months at −80 °C in TSX Universal Series ULT freezers (Thermo Fisher Scientific, Waltham, MA, USA). Details of the sample collection and processing were described previously [10]. Following the manufacturer’s protocol, RNA was extracted using the Zybio-Nucleic Acid Isolation System EXM 3000 (Zybio Inc., Chongqing, China). The resulting RNA extracts were then aliquoted and maintained at −80 °C in the same freezer model until further processing. To detect bocavirus, multiplex real-time reverse transcription polymerase chain reaction (RT-PCR) was performed at a centralized laboratory using the VIASURE Respiratory Panel III (Certest Biotec, S.L., Zaragoza, Spain). Comprehensive microbiological methods have been detailed in previous literature [10].

### 2.4. Power/Sample Size

The positivity rate for HBoV ranged from 10.0% to 18.3% based on previous studies from Jordan [7,8,14]. Regional data from Saudi Arabia revealed lower rates of 1.6% among 11,709 children 0 to 14 years old admitted to a children’s hospital with predominant respiratory symptoms during the study period [3]. Therefore, we expected 1.6% to 20% of the subjects to be positive for HBoV when calculating the sample size. A total of 1000 subjects who met the above clinical criteria were enrolled in the study.

### 2.5. Statistical Methods

Statistical analysis of the data was conducted using SPSS version 23. Descriptive statistics, specifically Student’s *t*-test and the chi-squared test, were employed to analyze and compare various categorical variables. These variables included demographic information, patient characteristics, relevant risk factors, and vaccination specifics.

To determine which factors might predict a positive HBoV test result, the likelihood of complications from HBoV, the likelihood of influenza positivity, or the likelihood of at least one complication, a logistic regression analysis was performed.

A patient was considered to have “at least one complication” if they experienced any of the following adverse outcomes: death (mortality), respiratory failure, a secondary bacterial or fungal infection (coinfection), respiratory distress severe enough to require supplemental oxygen (either through invasive or non-invasive means), pneumonia confirmed by X-ray, or cardiovascular issues (such as heart failure, bradycardia, or other related complications).

### 2.6. Case Report Form (CRF)

Parents/guardians of eligible patients were asked to provide consent for participation after being informed about the study details.

### 2.7. The Interview Forms Included Five Sections, as Described Previously [10]

1. Background, demographic, and societal data for patients and parents.
2. Medical history covering birth history, current medical conditions, and use of regular medications.
3. Presenting symptoms and signs: Details about clinical presentations and their duration, along with clinical manifestations and complications, were obtained.
4. Laboratory findings: This included white blood cells (WBC) and differential, blood gas, viral PCR results, chest X-ray findings on arrival, pharyngeal swab, and bacterial or fungal coinfections.

All medicines utilized during the admission, and those prescribed at discharge, were recorded.

### 2.8. Ethics

This study was conducted in accordance with the Declaration of Helsinki. Further analysis of the stored samples was approved by the Institutional Review Committee (IRC) of the Mutah University Faculty of Medicine Ethics Committee (Reference Number: 912023, dated 20 November 2023). The original project’s IRB approval was obtained from the Institutional Review Committee (IRC) for the Ministry of Health, IRB/REC/2022/295, dated 14 September 2022.

## 3. Results

### 3.1. Inpatient Data

A total of 1000 children were enrolled in the study, with an average age of 17.10 months (SD: 16.57) and a median age of 9.68 months (Q1–Q3: [3.13–29.83] months). The sample was slightly male-dominant, with nearly 60% of participants being male. Children came from both rural and urban communities, with 67.3% residing in rural areas, and were distributed evenly across the four participating cities: Amman 25.0%, Zarqa 25.0%, Irbid 25.0%, and Karak. Most were born at full term (84.3%), and around half were delivered by caesarean section (46.5%). About one in four had been admitted to a neonatal intensive care unit (24.3%), and a smaller proportion had required assisted ventilation 13.7%). Feeding practices varied: about one-third of children had been exclusively breastfed (33.5%), another third had received a combination of breast milk and formula (32.1%), and the final third were exclusively formula-fed (33.4%). Environmental exposures were also common; nearly one in four children lived in homes where smoking took place indoors, and household overcrowding affected roughly one in six families. Chronic health conditions were relatively uncommon, though a small proportion of children had asthma, congenital heart disease, or other congenital conditions.

Of the 1000 patients included in the analysis, 48 (4.8%) tested positive for HBoV. Positivity was highest in January 2023 (15.5%) compared with November 2022 (9.7%), December 2022 (11.0%), February 2023 (10.0%), and March 2023 (2.8%) (Supplementary Table S1). The mean age of HBoV-positive patients was 15.3 months (SD 16.4) compared with 17.2 months (SD 16.6) in the negative group. No significant difference was observed by sex (male: 5.6% positive; female: 3.6%). Geographically, the HBoV positivity rate was highest in Zarqa (6.4%), Irbid (6.0%), and Karak (5.2%), compared with Amman (1.6%; *p* = 0.049). Smoking inside the household was associated with a higher positivity rate (7.5% vs. 4.0%; *p* = 0.033) (Supplementary Table S2).

### 3.2. Clinical Symptoms

Fever (100%), cough (100%), and rhinorrhea (56.3%) were the most frequently reported symptoms among patients with HBoV (Supplementary Table S3). Other frequent symptoms included breathlessness (47.9%), respiratory crackles (58.3%), and low activity level (45.8%) (Table 1). Hypoxia/cyanosis was more common in coinfected patients (37.1%) compared with HBoV-only cases (7.7%; *p* = 0.046) (Supplementary Table S4). Duration of symptoms was generally similar between groups, except for apnea (>10 s), which lasted longer in HBoV-positive patients compared with HBoV-negative cases (mean 0.88 vs. 0.19 days; *p* = 0.003) (Supplementary Table S5). Supplementary Table S6 presents a comparative analysis of symptom duration between patients who were positive for human bocavirus (HBoV) alone and those who tested positive for HBoV in conjunction with co-infections. The analysis revealed no statistically significant differences in symptom duration between the two patient groups.

### 3.3. Clinical Findings

Infiltrates were present on chest X-ray in 62.5% of HBoV-positive patients compared with 64.8% of HBoV-negative patients (Table 1). Wheezing was reported in 64.6% of positive cases compared with 56.2% of negative cases (Table 1). Pneumothorax/atelectasis was rare but more frequent in positive cases (2.1% vs. 0.2%; *p* = 0.021). ICU admission was required for 12.5% of positive cases compared with 9.5% of negative cases (Table 1). Furthermore, a low activity level was significantly more frequent in HBoV-only patients (61.5%) than in coinfected patients (20.0%; *p* = 0.006) (Table 2).

### 3.4. Predictors of Positivity

Factors independently associated with HBoV positivity included household smoking (OR 2.87; 95% CI 1.51–5.49; *p* = 0.001) and residence in Zarqa (OR 6.98; 95% CI 2.16–22.54; *p* = 0.001), Irbid (OR 7.34; 95% CI 2.18–24.72; *p* = 0.001), or Karak (OR 6.97; 95% CI 2.02–24.06; *p* = 0.002) compared with Amman. A more extended hospital stay was also associated with HBoV positivity (OR 1.099 per additional day; 95% CI 1.004–1.20; *p* = 0.04) (Table 3).

### 3.5. Predictors of Complications

A multivariate logistic regression model was used to identify predictors of complications among the study population (*N* = 1000). HBoV infection showed an odds ratio (OR) of 1.623 (95% CI, 0.856–3.077; *p* = 0.138), indicating HBoV infection was not associated with a higher risk of complications.

A younger age was a strong predictor of complications, with each additional month of age decreasing the odds of complications (OR 0.953; 95% CI 0.943–0.963; *p* < 0.001). Female patients (OR 1.573; 95% CI 1.184–2.088; *p* = 0.002) had an increased risk of complications.

Among clinical conditions, asthma was significantly associated with complications (OR, 2.066; 95% CI, 1.086–3.926; *p* = 0.027) (Table 4).

## 4. Discussion

The positivity rate of HBoV of 4.8% among hospitalized children with respiratory symptoms younger than 5 years is comparable to data from the Middle East and globally during the COVID-19 transitional period and the post-COVID-19 era [7,14]. A study in Sri Lanka detecting HBoV in children with acute respiratory infection (ARI) reported prevalence rates of 4.94% in 2022 and 5.04% in 2023 [15]. In a study from the Middle East by Abdelqader et al. (2021) [7], the incidence of HBoV in pediatric respiratory tract infections ranged from 56.8% in Egypt to 0% in Iran. The discrepancy in results in the Middle East could be attributed to differences in testing techniques, sample collection methods, and viral detection methods, as well as staff experience, varying inclusion criteria, differing definitions of respiratory illness, and varying proportions of participants across age bands. Previous studies in Jordan before the COVID-19 pandemic reported varying HBoV positivity rates. For instance, Kaplan et al. reported rates ranging from 14% in December 2003 to 37% in April 2004 [8], while AL-Rousan et al. found a positivity rate of 9.1% in 2007 [14]. The most recent study by Awad et al. (2020) identified an HBoV incidence of only 2.5% in 2016 [7,8,14,16].

Data from the Middle East on HBoV positivity revealed wide variation in positivity and clinical burden [7,17]. Variations in HBoV prevalence in these populations are influenced by several factors, including the country’s geographic location, the clinical diagnosis of the population under study, the type of sample, the detection technique used, the age group of the population, and the season of the virus outbreak [18,19].

The highest rate of infection in our study was in January 2023 (15.5%). This is in line with other studies from Jordan, which indicated that the majority of HBoV infections (45%) are found between January and April, when respiratory illnesses are most common in Jordan [14]. There have been published reports of seasonal HBoV incidence from many nations. HBoV was found in Sweden and France during the winter and spring [20]; however, it was found more frequently between September and February in Hong Kong [21], although in Germany and Canada, no discernible seasonal predominance was seen [22].

Our population’s HBoV disease risk variables were comparable to those found in previous studies. This included younger age, multiple births, male sex, poor parental education and socioeconomic position, daycare attendance, young siblings, parental smoking, and a family history of asthma or atopy. The association of HBoV positivity with rural areas in our study is consistent with previous studies [14]. This is due to environmental, socioeconomic, and infrastructural factors in rural areas, increasing the risk of respiratory infections. Moreover, studies have revealed that a high rate of parental smoking inside the household is strongly associated with HBoV infection amongst children less than 5 years old [3,14].

Fever (100%), cough (100%), and rhinorrhea (56.3%) are the most common clinical respiratory symptoms linked to HBoV infection [14,21]. In the current study, additional common symptoms were low activity level (45.8%), respiratory crackles (58.3%), and dyspnea (47.9%). The same common clinical signs and symptoms associated with an HBoV infection were present in Jordan in the study by Al-Rousan et al. (2011) [14] including fever, cough, and rhinorrhea. The severity of cases attributed to coinfections, which involve one or more pathogens, was observed in 3.5% (*n* = 35) of participants, who had positive results for influenza virus and RSV, respectively. Co-infected patients presented with a more severe clinical picture, including more severe signs and clinical findings, as well as a longer duration of hospital stay. Many other studies noted severe cases amongst patients with viral and bacterial co-infections [23,24,25].

In our study, a previous diagnosis of asthma was associated with an increased risk of complications with an HBoV infection. This is consistent with another study that found that a personal or family history of atopy was associated with more severe HBoV [26].

Overall, the epidemiology, clinical presentation, and complications for children infected with HBoV in this study are similar to those previously reported from across the MENA region and globally [7].

Despite having a broad sample from several Jordanian locations, this study has several drawbacks. The study was based on RSV seasonality, and this report presents further analysis of the samples for HBoV. HBoV is known to persist in the respiratory tract for weeks to months with high asymptomatic carriage rates. It is recommended that future studies use quantitative PCR (e.g., a viral load threshold) or serology (e.g., IgM) to distinguish active infection from carriage.

HBoV seasonal changes and annual incidence data were not available. Gastrointestinal symptoms were not assessed thoroughly in this study because it was focused on respiratory symptoms. We propose further studies on HBoV round-year surveillance, including annual positivity rate assessments and a comprehensive evaluation of gastrointestinal and other clinical manifestations of HBoV infections.

This report and others published previously [10,11,12,13] indicate that respiratory infections create a major burden on the health of children younger than the age of five in Jordan. These reports provide baseline clinical and epidemiological data for future preventive measures for these infections. It was not feasible to combine them in one report due to the need for detailed information on each virus and to allow comparison with previous data from Jordan and globally. On the other hand, details about the presence of coinfections were presented in these reports.

## 5. Conclusions

Although the positivity rate for HBoV in children under five years old in Jordan is lower than before the COVID-19 pandemic, the rate of 4.8%, along with significant clinical manifestations in many children, highlights the importance of HBoV as one of the common respiratory pathogens leading to morbidity in Jordan. This study will contribute to global data on HBoV burden and stimulate future research into HBoV prevention in Jordan and worldwide.

## Figures and Tables

**Table 1:** Clinical findings in negative vs. positive bocavirus cases.

Clinical Finding		Negative (*N* = 952), *n* (%)	Positive (*N* = 48), *n* (%)	*p*-Value
Chest X-ray infiltrate		617 (64.8)	30 (62.5)	0.744
White Blood Cells Count	WBC > 10.0 × 10^9^/L	13 (1.4)	0 (0.0)	0.634
	WBC < 4.0 × 10^9^/L	338 (35.6)	19 (39.6)	—
	WBC 4–10 × 10^9^/L	599 (63.1)	29 (60.4)	—
Other Clinical Manifestations:				
Cardiovascular		10 (1.1)	0 (0.0)	0.475
Low activity level		283 (29.7)	15 (31.3)	0.822
Apnea > 10 s		10 (1.1)	0 (0.0)	0.475
Dehydration		221 (23.2)	12 (25.0)	0.775
Hypoxia (SpO_2_ < 92%)		225 (23.6)	9 (18.8)	0.436
Subcostal/intercostal retractions		407 (42.8)	24 (50.0)	0.323
Wheeze		535 (56.2)	31 (64.6)	0.253
Tachypnea		485 (51.1)	29 (60.4)	0.2
Cyanosis		72 (7.6)	6 (12.5)	0.213
Pneumothorax/atelectasis		2 (0.2)	1 (2.1)	0.021
Acute respiratory distress		274 (28.8)	8 (16.7)	0.069
Nasal flaring		18 (1.9)	0 (0.0)	0.336
ICU admission		90 (9.5)	6 (12.5)	0.484
Oxygen need (non-invasive ventilation)		57 (6.0)	2 (4.2)	0.601
Oxygen need (invasive ventilation)		9 (1.0)	0 (0.0)	0.499

Values are *n* (%). *p*-values are chi-square tests between negative and positive bocavirus participants. Significant at *p* < 0.05.

**Table 2:** Clinical findings in bocavirus-positive only vs. bocavirus-positive with coinfection.

Clinical Finding		Positive Only (*N* = 13), *n* (%)	Positive + Coinfection (*N* = 35), *n* (%)	*p*-Value
Chest X-ray infiltrate		10 (76.9)	20 (57.1)	0.208
White Blood Cells Count	WBC > 10.0 × 10^9^/L	0 (0.0)	0 (0.0)	0.571
	WBC < 4.0 × 10^9^/L	6 (46.2)	13 (37.1)	—
	WBC 4–10 × 10^9^/L	7 (53.9)	22 (62.9)	—
Other Clinical Manifestations:				
Cardiovascular		0 (0.0)	0 (0.0)	—
Low activity level		8 (61.5)	7 (20.0)	0.006
Apnea > 10 s		0 (0.0)	0 (0.0)	—
Dehydration		2 (15.4)	10 (28.6)	0.348
Hypoxia (SpO_2_ < 92%)		1 (7.7)	8 (22.9)	0.232
Subcostal/intercostal retractions		6 (46.2)	18 (51.4)	0.745
Wheeze		7 (53.9)	24 (68.6)	0.343
Tachypnea		9 (69.2)	20 (57.1)	0.447
Cyanosis		1 (7.7)	5 (14.3)	0.539
Pneumothorax/atelectasis		1 (7.7)	0 (0.0)	0.097
Acute respiratory distress		2 (15.4)	6 (17.1)	0.884
Nasal flaring		0 (0.0)	0 (0.0)	—
ICU admission		2 (15.4)	4 (11.4)	0.713
Oxygen need (non-invasive ventilation)		0 (0.0)	2 (5.7)	0.379
Oxygen need (invasive ventilation)		0 (0.0)	0 (0.0)	—

Values are *n* (%). *p*-values are chi-square tests between bocavirus-positive only and bocavirus-positive with coinfection participants. Significant at *p* < 0.05. Coinfection refers to cases positive for respiratory syncytial virus, influenza virus, human rhinovirus, human adenovirus, parainfluenza, or coronavirus, also positive for bocavirus.

**Table 3:** Binary multivariate logistic regression analysis of factors associated with bocavirus-positive results across all age groups.

Factor	Coef.	Odds Ratio	St.Err.	*p*-Value	95% CI
**Total length of hospital stays**	0.095	1.099	0.05	0.04	1.004–1.2
**Smoking inside house by parents**	1.055	2.87	0.95	0.001	1.51–5.49
**City**					
Zarqa	1.943	6.98	4.18	0.001	2.16–22.54
Irbid	1.993	7.34	4.55	0.001	2.18–24.72
Karak	1.941	6.97	4.41	0.002	2.02–24.06
**Constant**	−5.336	0.005	0.003	<0.001	0.001–0.018

The reference category in all binary variables was assigned to “No” as a reference group. For categorical variables, city: Amman.

**Table 4:** Binary multivariate logistic regression analysis of factors associated with the presence of complications across all age groups.

Factor	Coef.	Odds Ratio	St.Err.	*p*-Value	95% CI
Bocavirus	0.484	1.623	0.53	0.138	0.856–3.077
Influenza	−1.293	0.275	0.097	<0.001	0.137–0.551
Age (month)	−0.048	0.953	0.005	<0.001	0.943–0.963
Gender	0.453	1.573	0.227	0.002	1.184–2.088
Total length of hospital stay	0.105	1.11	0.026	<0.001	1.06–1.163
Asthma	0.725	2.066	0.677	0.027	1.087–3.926
Constant	−0.491	0.612	0.093	0.001	0.454–0.826

The reference category in all binary variables was assigned to “No” as a reference group. For categorical variables, gender: male.

## Data Availability

The original contributions presented in the study are included in the article/Supplementary Material; further inquiries can be directed to the corresponding author.

## Supplementary Materials

The following supporting information can be downloaded at: https://www.mdpi.com/article/10.3390/pathogens15050515/s1. Table S1: Bocavirus positivity by month; Table S2: Investigating demographic Factors Associated with Bocavirus results; Table S3: Presence of Symptoms Among Negative vs Positive Bocavirus Cases; Table S4: Presence of Symptoms Among Bocavirus-Positive Only vs Bocavirus-Positive With Coinfection; Table S5: Duration of symptoms by Bocavirus positivity; Table S6: Duration of symptoms across Bocavirus positive only and bocavirus with coinfection.

## Abbreviations

The following abbreviations are used in this manuscript:

HBoV	Human bocavirus
MENA	Middle East and North Africa
RSV	Respiratory syncytial virus
SARS-CoV-2	Severe acute respiratory syndrome coronavirus 2
NP	Nasopharyngeal
RT-PCR	Reverse transcription polymerase chain reaction
ULT	Ultra-low temperature
SPSS	Statistical Package for the Social Sciences
CRF	Case report form
WBC	White blood cell
IRC	Institutional Review Committee
IRB	Institutional Review Board
ICU	Intensive care unit
OR	Odds ratio
CI	Confidence interval
SD	Standard deviation
Q1–Q3	First quartile to third quartile
