# Supplementary material for: National Cross-Sectional Study Assessing the Positivity Rate and Clinical Manifestations of Human Bocavirus Respiratory Infections Among Hospitalized Children Under 5 Years of Age in Jordan
Source: Pathogens. 2026 May 12;15(5):515. doi: 10.3390/pathogens15050515 (PMC13209634; doi:10.3390/pathogens15050515)
Supplement: Supplementary file 1 [file pathogens-15-00515-s001.zip › pathogens-4248112-supplementary.pdf]

Supplementary Materials:

Supplementary Table S1. Bocavirus positivity by month

			Bocavirus		Total	P-value*
			NO	YES		
Date month	Nov-2022	Count	289	31	320	0.182
		% within Month	90.3%	9.69%	100%	
	Dec-2022	Count	406	50	456	
		% within Month	89.0%	11.0%	100%	
	Jan-2023	Count	125	23	148	
		% within Month	84.5%	15.5%	100%	
	Feb-2023	Count	36	4	40	
		% within Month	90.0%	10.0%	100%	
	Mar-2023	Count	35	1	36	
		% within Month	97.2%	2.78%	100%	

*Chi-square, statistically significant at P < 0.05.

Supplementary Table S2: Investigating demographic Factors Associated with Bocavirus results.

Characteristic	Bocavirus				P-value*
	Negative (N=952)		Positive (N=48)		
	Count	Row (N %)	Count	Row (N %)	
**Age in months, mean (SE)**	17.2(16.56)		15.3(16.4)		0.459
**Age ≤ 6 months**					
NO (n=623)	599	96.2%	24	3.80%	
YES (n=377)	353	93.6%	24	6.40%	0.072
**Gender, n (%)**					
**Male patients (n=586)**	553	94.4%	33	5.60%	0.143
**Female patients (n=414)**	399	96.4%	15	3.60%	
**City, n (%)**					
· Amman (n=250)	246	98.4%	4	1.60%	0.049
· Zarqa (n= 250)	234	93.6%	16	6.40%	
· Irbid(n=250)	235	94.0%	15	6.00%	
· Karak (n=250)	237	94.8%	13	5.20%	

**Living area, n (%)**						
**Rural (n= 673)**		642	95.4%	31	4.61%	0.681
**Urban(n=327)**		310	94.8%	17	5.20%	
**Preterm or Full term**	full term	800	94.90%	43	5.10%	0.302
	preterm	152	96.82%	5	3.18%	
**Delivery**	Normal VD, (n=535)	509	95.1%	26	4.90%	0.924
	Caesarean (n= 465)	443	95.3%	22	4.70%	
**Meconium-stained liquor**	NO (n=982)	934	95.1%	48	4.90%	
	YES (n=18)	18	100%	0	0.00%	0.336
**NICU**	NO (n=757)	718	94.8%	39	5.20%	
	YES (n=243)	234	96.3%	9	3.70%	0.358
**NICU ventilation**	NO (n=863)	822	95.3%	41	4.70%	0.855
	YES (n=137)	130	94.9%	7	5.11%	
**Surfactant Given**	NO (n=836)	796	95.2%	40	4.78%	0.959
	YES (n=164)	156	95.1%	8	4.88%	
**Breastfed**	NO (n=334)	311	93.1%	23	6.90%	
	Exclusive (n=345)	333	96.5%	12	3.50%	0.087
	Mixed (n=321)	308	95.9%	13	4.10%	
**Mother smoking during pregnancy**	NO (n=912)	908	95.4%	44	4.62%	0.241
	YES (n=48)	44	91.7%	4	8.30%	
**Patient on special milk/diet**	NO (n=966)	918	95.0%	48	5.00%	0.183
	YES (n=34)	34	100%	0	0.00%	
**Overcrowding: (more than 3 more than three people per habitable room)**	NO (n=837)	793	94.7%	44	5.30%	
	YES (n= 163)	159	97.6%	4	2.40%	0.126
**Parents smoking cigarettes or Shisha**	NO (n=277)	265	95.7%	12	4.30%	0.668
	YES (n=723)	687	95.02%	36	4.98%	
**Smoking inside home by parents or other households**	NO (n=772)	741	95.98%	31	4.02%	
	YES (n=228)	211	92.5%	17	7.50%	0.033

**Patient regular medications**	NO (n=813)	773	95.1%	40	4.9%	
	YES (n=187)	179	95.7%	8	4.30%	0.711
**Highest completed education of Father**	Primary school (n=136)	129	94.9%	7	5.10%	
	Secondary school (n=495)	471	95.2%	24	4.80%	
	Diploma school (n=80)	77	96.3%	3	3.70%	
	University BSc (n=236)	224	94.9%	12	5.10%	0.983
	University Postgraduate (n=53)	51	96.2%	2	3.80%	
**Highest completed education of Mother**	Primary school (n=109)	105	96.3%	4	3.70%	
	Secondary school (n=491)	464	94.5%	27	5.50%	
	Diploma school (n=89)	85	95.5%	4	4.49%	
	University BSc (n=284)	272	95.8%	12	4.23%	0.890
	University Postgraduate (n=27)	26	96.3%	1	3.70%	
**Chronic conditions:**						
**Asthma**	No (n=948)	903	95.3%	45	4.70%	0.737
	Yes (n= 52)	49	94.2%	3	5.80%	
**Bronchopulmonary dysplasia**	No (n=999)	951	95.2%	48	4.80%	0.822
	Yes (n=1)	1	100%	0	0.00%	
**Congenital heart disease**	No (n=958)	912	95.2%	46	4.80%	0.991
	Yes (n=42)	40	95.2%	2	4.80%	
**Neuromuscular disease**	No (n=986)	939	95.2%	47	4.80%	0.680
	Yes (n=14)	13	92.9%	1	7.14%	
**Other comorbidities****	No (n=888)	846	95.3%	42	4.70%	0.770
	Yes (n=112)	106	94.6%	6	5.40%	
**Cystic fibrosis**	No					
	Yes					
**Other congenital disease*****	No (n=988)	942	95.3%	46	4.70%	
	Yes (n=12)	10	83.3%	2	16.7%	0.053

**Eczema (Atopy)**	NO (n=977)	929	95.1%	48	4.90%	0.276
	YES (n=23)	23	100%	0	0.00%	
**Patient Chronic Conditions**	NO (n=782)	744	95.1%	38	4.90%	0.868
	YES (n=218)	208	95.4%	10	4.60%	
**Patient attending kindergarten or day care**	NO (n=909)	866	95.3%	43	4.70%	0.745
	YES (n=91)	86	94.5%	5	5.60%	
**Parent with history of atopic eczema**	NO (n=919)	872	94.9%	47	5.10%	0.117
	YES (n=81)	80	98.8%	1	1.23%	
**Siblings attending kindergarten or daycare**	NO (n=803)	763	95.0%	40	5.00%	0.588
	YES (n=197)	189	95.9%	8	4.10%	
**Parent with history of asthma**	NO (n=916)	872	95.2%	44	4.80%	0.986
	YES (n=84)	80	95.2%	4	4.80%	
**Siblings with history of asthma**	NO (n=918)	875	95.3%	43	4.70%	0.566
	YES (n=82)	77	93.9%	5	6.10%	
**Siblings <5 years living in the same household**	NO	389	95.3%	19	4.70%	
	YES	563	95.1%	29	4.90%	0.861

*Chi-square test, statistically significant at p< 0.05. For 'Age' the t-test was used.

** Other comorbidities: including congenital heart disease, Ventricular Septal Defect (VSD), Antenatally Diagnosed Congenital Pulmonary Airway Malformation (CPAM) Type 1 with extra lobar sequestration, and Complete Atrioventricular (AV) Canal, along with Atrial Septal Defect (ASD) and VSD leading to heart failure. Additionally, Down syndrome, Global Developmental Delay (GDD), and epilepsy. Other conditions include Reactive Airway Disease (RAD), Retinopathy of Prematurity (ROP), renal anomalies, seizures and Glucose-6-Phosphate Dehydrogenase Deficiency (G6PD).

***In terms of other congenital diseases, these included: chest deformity, Gastroesophageal Reflux Disease (GERD), Pierre Robin Syndrome, horseshoe kidney, laryngomalacia, single kidney, single lung, hydrocephalus, and Vesicoureteral Reflux (VUR).

Supplementary Table s3 . Presence of Symptoms Among Negative vs Positive Bocavirus Cases.

Symptom	Negative (N = 952), n (%)	Positive (N = 48), n (%)	*P* value
Fever	940 (98.7)	48 (100.0)	0.434
Cough	890 (93.5)	48 (100.0)	0.068
Sore throat	197 (20.7)	7 (14.6)	0.305
Rhinorrhea	510 (53.6)	27 (56.3)	0.717
Nasal congestion	437 (45.9)	17 (35.4)	0.155
Poor feeding	475 (49.9)	18 (37.5)	0.094
Hypoxia/cyanosis	266 (27.9)	14 (29.2)	0.854

Breathlessness	405 (42.5)	23 (47.9)	0.463
Respiratory crackles	538 (56.5)	28 (58.3)	0.804
Apnea >10 sec	10 (1.1)	0 (0.0)	0.475
Low activity level	515 (54.1)	22 (45.8)	0.263
Tachypnea	327 (34.4)	15 (31.3)	0.658
Post-tussive vomiting	330 (34.7)	11 (22.9)	0.094

Percentages calculated as n/N × 100. *P* values are based on chi-square tests between negative and positive Bocavirus participants. Significant at $p < .05$.

Supplementary Table S4. Presence of Symptoms Among Bocavirus-Positive Only vs Bocavirus-Positive With Coinfection.

Symptom	Positive Only (N = 13), n (%)	Positive + Coinfection (N = 35), n (%)
Fever	13 (100.0)	35 (100.0)
Cough	13 (100.0)	35 (100.0)
Sore throat	2 (15.4)	5 (14.3)
Rhinorrhea	8 (61.5)	19 (54.3)
Nasal congestion	7 (53.9)	10 (28.6)
Poor feeding	6 (46.2)	12 (34.3)
Hypoxia/cyanosis	1 (7.7)	13 (37.1)
Breathlessness	6 (46.2)	17 (48.6)
Respiratory crackles	8 (61.5)	20 (57.1)
Apnea >10 sec	8 (61.5)	14 (40.0)
Low activity level	8 (61.5)	14 (40.0)
Tachypnea	6 (46.2)	9 (25.7)
Post-tussive vomiting	2 (15.4)	9 (25.7)

Percentages calculated as n/N × 100. *P* values are based on chi-square tests between Bocavirus-positive onl[illegible] Bocavirus-positive with coinfection participants. Significant at $p < .05$. Coinfection refers to cases positive [illegible] Respiratory Syncytial virus, influenza virus, human rhinovirus, human adenovirus, parainfluenza, or coronavirus, also positive for Bocavirus.

Supplementary Table S5. Duration of symptoms by Bocavirus positivity..

	Bocavirus result	

Symptoms	Negative			Positive			P-value*
	Mean	SD	[95% CI]	Mean	SD	[95% CI]	
**Fever (days)**	3.94	3.69	3.71- 4.17	4.35	4.54	3.04 -5.67	0.45
**Cough (days)**	5.45	5.65	5.09- 5.80	5.56	4.63	4.22- 6.90	0.89
**Sore throat (days)**	0.97	2.7	0.80- 1.14	1.21	4.54	-2.64	0.57
**Rhinorrhea (days)**	3.03	4.31	2.76 3.31	3.48	5.32	1.93 - 5.03	0.49
**Nasal congestion (days)**	2.58	4.58	2.29- 2.87	2.08	3.73	1.00- 3.16	0.46
**Poor Feeding (days)**	1.8	2.79	1.62- 1.97	1.08	1.57	0.63- 1.53	0.08
**Hypoxia/Cyanosis (days)**	1.01	3.13	0.81 1.20	1.39	4.49	0.09- 2.69	0.41
**Breathlessness (days)**	1.64	2.8	1.46- 1.82	1.67	2.19	1.03- 2.30	0.94
**Respiratory crackles (days)**	2.34	3.21	2.14- 2.54	2.65	4.62	1.30- 3.98	0.53
**Low activity level (days)**	1.98	2.83	1.80- 2.16	1.35	1.76	0.84 -1.86	0.132
**Tachypnea (days)**	1.14	2.17	0.997- 1.27	1.13	2.05	0.53- 1.71	0.974
**Post Tussive Vomiting (days)**	1.07	2.07	0.94 1.20	0.67	1.48	0.24 -1.10	0.183

* T-test statistically significant at $p < 0.05$.

Supplementary Table S6 - Duration of symptoms across Bocavirus positive only and bocavirus with coinfection

	Bocavirus result						
Symptoms	Bocavirus Only			Bocavirus with coinfection**			P-value*
	Mean	SD	[95% CI]	Mean	SD	[95% CI]	
**Fever (days)**	4.23	4.04	1.79 - 6.67	4.4	4.76	2.76 - 6.03	0.91
**Cough (days)**	6.00	4.65	3.19 - 8.81	5.4	4.67	3.79 - 7.00	0.69
**Sore throat (days)**	0.38	1.39	-0.45 - 1.22	1.51	5.24	-0.28 - 3.31	0.45
**Rhinorrhea (days)**	4.08	4.87	1.13 - 7.02	3.26	5.54	1.35 - 5.16	0.64
**Nasal congestion (days)**	3.54	5.08	0.47 - 6.61	1.54	3.00	0.51 - 2.57	0.09
**Poor Feeding (days)**	1.54	1.94	0.36 - 2.71	0.91	1.40	0.43 - 1.39	0.22
**Hypoxia/Cyanosis (days)**	0.08	0.28	-0.09 - 0.24	1.88	5.19	0.103 - 3.66	0.22
**Breathlessness (days)**	1.31	2.25	-0.05 - 2.67	1.8	2.18	1.05 - 2.55	0.49
**Respiratory crackles (days)**	1.69	2.02	0.47 - 2.91	3.00	5.25	1.19 - 4.81	0.39
**Low activity level (days)**	1.92	2.06	0.68 - 3.7	1.14	1.61	0.59 - 1.69	0.17
**Tachypnea (days)**	1.38	2.06	0.14 - 2.63	1.03	2.06	0.32 1.74	0.59
**Post Tussive Vomiting (days)**	0.61	1.94	-0.56 1.78	0.68	1.30	0.24 1.13	0.88

* T-test statistically significant at $p < 0.05$.

Coinfection refers to cases positive for Respiratory Syncytial virus, influenza virus, human rhinovirus, human adenovirus, parainfluenza, or coronavirus, also positive for Bocavirus.
